# NSP16 2′-O-MTase in Coronavirus Pathogenesis: Possible Prevention and Treatments Strategies

**DOI:** 10.3390/v13040538

**Published:** 2021-03-24

**Authors:** Li-Jen Chang, Tsung-Hsien Chen

**Affiliations:** 1 Division of Gastroenterology and Hepatology, Department of Internal Medicine, Ditmanson Medical Foundation Chia-Yi Christian Hospital, Chiayi 60002, Taiwan; cych07235@gmail.com; 2 Department of Internal Medicine, Ditmanson Medical Foundation Chia-Yi Christian Hospital, Chiayi 60002, Taiwan

**Keywords:** coronavirus, nonstructural protein 16, 2′-O-MTase, prevention, treatment

## Abstract

Several life-threatening viruses have recently appeared, including the coronavirus, infecting a variety of human and animal hosts and causing a range of diseases like human upper respiratory tract infections. They not only cause serious human and animal deaths, but also cause serious public health problems worldwide. Currently, seven species are known to infect humans, namely SARS-CoV-2, MERS-CoV, SARS-CoV, HCoV-229E, HCoV-NL63, HCoV-OC43, and HCoV-HKU1. The coronavirus nonstructural protein 16 (NSP16) structure is similar to the 5′-end capping system of mRNA used by eukaryotic hosts and plays a vital role in evading host immunity response and protects the nascent viral mRNA from degradation. NSP16 is also well-conserved among related coronaviruses and requires its binding partner NSP10 to activate its enzymatic activity. With the continued threat of viral emergence highlighted by human coronaviruses and SARS-CoV-2, mutant strains continue to appear, affecting the highly conserved NSP16: this provides a possible therapeutic approach applicable to any novel coronavirus. To this end, current information on the 2′-O-MTase activity mechanism, the differences between NSP16 and NSP10 in human coronaviruses, and the current potential prevention and treatment strategies related to NSP16 are summarized in this review.

## 1. Introduction

A high-incidence respiratory illness that is coronavirus disease 2019 (COVID-19) is caused by person-to-person transmission of the novel coronavirus, now termed severe acute respiratory syndrome coronavirus 2 (SARS-CoV-2) by the ICTV Coronaviridae Study Group on 12 February 2020, and was identified in December 2019 [1]. Since then, COVID-19 has become a global pandemic [2] and has led the World Health Organization (WHO) to declare a public health emergency [3]. The person-to-person transmission of coronaviruses is mainly achieved through close contact with respiratory droplets, direct contact with infected individuals, or contact with contaminated objects and surfaces. Currently, seven coronaviruses are known to infect humans. Four of these are very common (HCoV-229E, HCoV-NL63, HCoV-OC43, and HCoV-HKU1) and cause an upper respiratory disease with symptoms of the common cold [4,5]. The three other coronaviruses, severe acute respiratory syndrome coronavirus (SARS-CoV), SARS-CoV-2, and Middle East respiratory syndrome coronavirus (MERS-CoV), have appeared more recently and cause severe respiratory syndrome in humans [6]. SARS-CoV-2 has approximately 79% genome similarity with SARS-CoV and 50% genome similarity with MERS-CoV [7]. Its clinical manifestations include fever, cough, fatigue, shortness of breath, rhinorrhea, sneezing, sore throat, and occasionally watery diarrhea, nausea/vomiting, and abdominal pain [8,9].

SARS-CoV was first identified in 2002 and diagnosed in Southern China [10], with a case fatality rate of approximately 3% [11]. Then, MERS-CoV infected people with a high mortality rate of approximately 60% in the Middle East [12]. As of September 2019, the global case count for MERS-CoV was 2468 laboratory-confirmed cases, including 851 associated deaths and a case fatality rate of 34.4% [13]. Lastly, SARS-CoV-2 was first identified in December 2019 [1] and has since become a global pandemic [2]. As of 7 February 2021, 105,394,301 confirmed COVID-19 cases have been reported by WHO globally, including 2,302,302 deaths, thus having a case fatality rate of 2.18% [14]. However, compared with SARS-CoV and MERS-CoV, SARS-CoV-2 seems to be more contagious and spreads more effectively from person to person, and thus led to a global public health emergency. Furthermore, its higher transmission and pathogenicity can be attributed to genetic alterations in nonstructural proteins and different structures [15].

## 2. Coronaviruses

**Figure 1:**
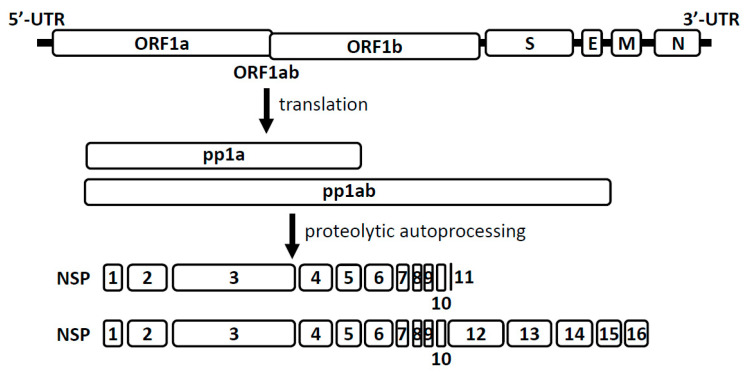
Genomic organization of representative human coronaviruses. E: envelope, M: membrane, N: nucleocapsid, NSP: nonstructural proteins, ORF: open reading frames, S: spike, UTR: untranslated region.

Coronaviruses are enveloped, positive-sense RNA viruses containing a single-stranded RNA genome with a 5′-terminal cap structure and 3′-polyadenylation, and can infect a wide variety of mammalian and avian species [16,17]. The genome organization for a coronavirus is: 5′-leader-UTR-replicase (open reading frames 1ab)-spike-envelope-membrane-nucleocapsid-3′UTR-polyA tail (Figure 1). Coronaviruses (order *Nidovirales*, family *Coronaviridae*, and subfamily *Orthocoronavirinae*) are divided into four genera: *Alphacoronavirus*, *Betacoronavirus*, *Gammacoronavirus*, and *Deltacoronavirus*, with the human coronaviruses under *Alphacoronavirus* (HCoV-229E and -NL63) and *Betacoronavirus* (MERS-, SARS-CoV, HCoV-OC43, and -HKU1).

The virus particles are formed by four major structural proteins: spike, envelope, membrane, and nucleocapsid. The envelope and membrane proteins with spike protein form a viral envelope, with the spike protein allowing the virus to attach to the host cell membrane, and the nucleocapsid protein holding the virus RNA genome [18]. The replicase polyprotein cleaves itself to form 16 nonstructural proteins (NSP1 to NSP16) [19], which form the replication–transcription complex localized in endoplasmic reticulum-derived membranes [20,21]. Among them, NSP10, NSP13, NSP14, and NSP16 are involved in mRNA capping, and NSP10 acts as an activation cofactor in a complex with NSP14 to proofread and edit newly synthesized RNA [22,23]. Additionally, the nonstructural RNA genome of RNA-dependent RNA polymerase (NSP12) function is needed for positive-sense viruses during viral genome replication [24]. The loss of the interaction between NSP10 and NSP16 leads to the inhibition of viral replication, which indicates that formation of the complex is an essential step in the life cycle of coronavirus [25].

## 3. Host Antiviral Immune Response

Patients infected with SARS-CoV, MERS-CoV, or SARS-CoV-2 usually develop acute lung injury, systemic inflammatory response syndrome, acute respiratory distress syndrome, and renal failure, eventually leading to death [26]. When coronaviruses invade lung epithelial cells and alveolar macrophages to produce viral RNA, this stimulates the infected cells to release cytokines and chemokines, thereby activating macrophages, dendritic cells, and others [27]. Cytokines play an important key role in driving the appearance of these clinical features and are also at the core of the development of inflammation [28,29]. Among them, host T helper 17 hyperinflammation responses are major contributors to cytokine storms, a hallmark of SARS-CoV-2 infections [30,31].

Coronaviruses activate the release of various pro-inflammatory cytokines in the host during the infection process, such as interleukin (IL)-1β, IL-2, IL-6, IL-7, granulocyte colony-stimulating factor, interferon, and tumor necrosis factor [32,33,34]. Subsequently, chemokines and cytokines are increasingly released from these cells, attracting more inflammatory cells to migrate from blood vessels to the site of inflammation. IL-1β and IL-6 are the main pro-inflammatory cytokines released during coronavirus infections [35], with the former enhancing the inflammatory response of bronchi and alveoli in patients with lung injury. At the same time, hepatocytes are stimulated by IL-1β and IL-6 to produce acute-phase proteins and activate the complement system, further increasing vascular permeability. The structural and nonstructural proteins of the coronaviruses can cause a delayed response of interferon, which promotes viral replication, increases the expression of viral pathogen-associated molecular patterns, and enhances the inflammatory response [36,37,38]. In mammals, the main antiviral innate immune response is an interferon-mediated response. Coronavirus NSP10 forms a complex with NSP16 during the coronavirus life cycle, and this immune evasion ability interferes with activation of the type-1 interferon (IFN1) response [39].

## 4. Coronavirus RNA Capping Mechanisms

Eukaryotic hosts utilize a 5′-terminal capping system to promote efficient nuclear export, robust translation, and enhanced stability of mRNA. In addition, mRNA capping also helps to distinguish between self and nonself RNA and can lead to initiation of the host immune response. However, many viruses that replicate in the cytoplasm of eukaryotes have evolved 2′-O-methyltransferase (2′O-MTase) to autonomously modify their mRNAs, thus being similar to the 5′-cap structures of higher eukaryote mRNAs that have ribose 2′-O-methylation [40]. Thus, such RNA modifications provide a molecular signature to avoid the host’s discrimination between self and nonself mRNA [41]. This special structure, 2′-O-methylation, allows viral mRNA to mimic that of humans and is critically involved in subverting the induction of IFN1 [41]. Thus, overcoming the host immune response is paramount to the success of any viral infection.

The usual viral RNA cap structure, the guanosine cap core structure, the N7 methylation, and the 2′-O-methylation respectively protect the 5′-triphosphate from activating the host innate immune response [42,43]; ensure viral replication through the enhancement of viral RNA translation [44]; elude the recognition of host RNA sensors such as retinoic-acid-inducible protein 1 (RIG-I), melanoma differentiation-associated protein 5 (MDA5), and interferon-induced protein with tetratricopeptide repeats (IFIT); and resist interferon-mediated antiviral response [41,45,46]. The elusion from host RNA sensors is important because RIG-I is a cytosolic pattern recognition receptor, responsible for the IFN1 response when the host is infected by an RNA virus [47], and MDA5 is an RIG-I-like receptor dsRNA helicase encoded by the IFIH1 gene, which can recognize viruses and regulate IFN1 gene transcription [48].

The mRNA cap for coronaviruses is completed by NSP16, an m7GpppA-specific, S-adenosyl-L-methionine (SAM)-dependent 2′-O-MTase [49,50]. The cap installation process is catalyzed by another viral protein, NSP10, as a zinc-binding protein that binds nonspecifically to viral RNA and stabilizes the SAM binding pocket in NSP16, forming a stable complex [51]. NSP10 binding provides support for the SAM-binding of NSP16 as well as extending the RNA-binding groove of the complex [52]. This complex interaction between NSP16 and NSP10 is required for stability and 2′-O-MTase activity. For example, SARS-CoV, NSP16, and NSP10 complexes selectively 2′-O-methylate the cap 0 (m7GpppN) RNA structure [53]. The 2′-O-MTase catalyzes methylation at the 2′-O-ribose of the first nucleotide of the RNA to generate cap 1 (m7GpppNm), thereby preventing recognition by either MDA5 or IFIT [52]. Thus, the NSP10- and NSP16-mediated 2′-O-methylation of coronavirus RNA prevents host recognition and decreases host immune response while the viral RNA is translated [39,49,54,55].

## 5. Comparison of the NSP16 and NSP10 of Various Human Coronaviruses

**Figure 2:**
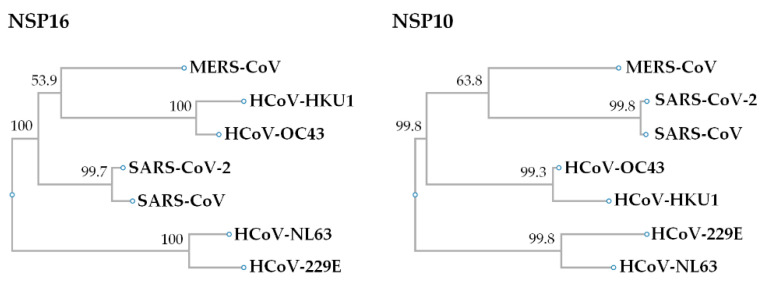
Phylogenetic analysis of the NSP16/NSP10 amino acid sequences of severe acute respiratory syndrome coronavirus 2 (SARS-CoV-2) with other human coronaviruses. The phylogenetic relationship of SARS-CoV-2 NSP16 and NSP10 to other human coronaviruses was constructed by the ETE3 method of CLUSTALW software. The bootstrap values are shown as percentages at the nodes. Human coronaviruses NCBI Reference Sequence Database (RefSeq) sequences from GenBank accession numbers: NC_045512 (SARS-CoV-2), NC_004718 (SARS-CoV), NC_019843 (MERS-CoV), NC_006213 (HCoV-OC43), NC_006577 (HCoV-HKU1), NC002645 (HCoV-229E), and NC005831 (HCoV-NL63).

**Figure 3:**
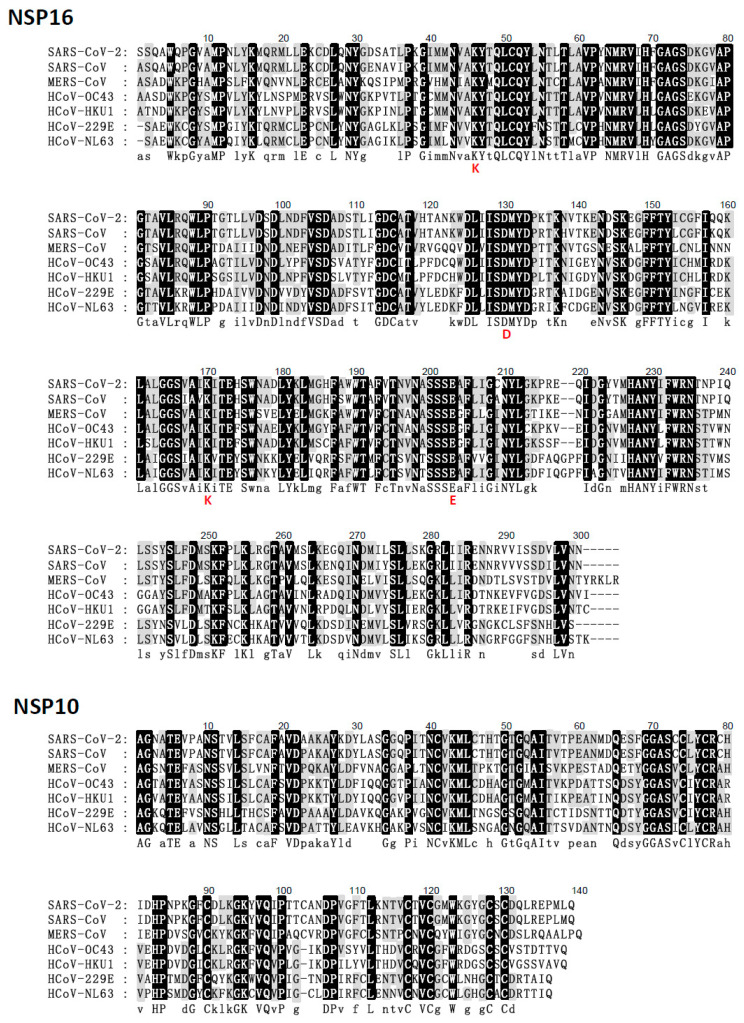
Multiple sequence alignment of the NSP16/NSP10 of SARS-CoV-2 with other human coronaviruses. Human coronaviruses NCBI Reference Sequence Database (RefSeq) sequences from GenBank accession numbers for protein sequence alignment analysis are as follows (http://www.ncbi.nim.nih.gov/genebank/) (accessed on 8 January 2021): NC_045512 (SARS-CoV-2), NC_004718 (SARS-CoV), NC_019843 (MERS-CoV), NC_006213 (HCoV-OC43), NC_006577 (HCoV-HKU1), NC002645 (HCoV-229E), and NC005831 (HCoV-NL63).** The KDKE motif is shown in red letters.

The presence of these host and virus effectors suggests the importance of NSP16 (2′-O-methylation) as a virulence determinant during coronavirus infection [56,57]. For example, the genetic destruction of SARS-CoV NSP16 significantly reduces the synthesis of viral RNA by ten-fold [58]. Thus, the ablation of NSP16 activity should trigger an immune response to coronavirus infection and limit the pathogenesis [41,45]. Phylogenetic analysis to determine the evolution of human coronaviruses using NCBI RefSeq NSP16 sequences indicates that genomic SARS-CoV-2 and SARS-CoV formed one cluster (99.7% identity), HCoV-HKU1 and HCoV-OC43 formed one cluster (100% identity) and formed a cluster with MERS-CoV (53.9% identity), while HCoV-NL63 and HCoV-229E formed another cluster (100% identity) (Figure 2). The SARS-CoV-2 and SARS-CoV NSP16 proteins have 298 residues, and that of other human coronaviruses have 298–303 residues (Figure 3, Table 1). However, the amino acid composition of NSP16 between different human coronaviruses is only slightly different (Table 1). Besides, NSP16 utilizes SAM MTase protein fold [59,60] with slight variations [50,52]. The SAM MTase binding sites (K46, D130, K170, and E203, the KDKE motif) are highly conserved among all human coronavirus sequences examined (Figure 3). The SAM MTase binding site is near the SAM methyl group catalytically transferred to the 2′-O sugar [50,52,54,55,61]. SAM binding promotes the assembly of the enzymatically active NSP10/NSP16 complex, which converts cap 0 RNA into cap 1 [62]. Because of this, mutation of the KDKE motif has been shown to ablate 2′-O-MTase activity and attenuate various aspects of infection [41,63,64].

**Figure 4:**
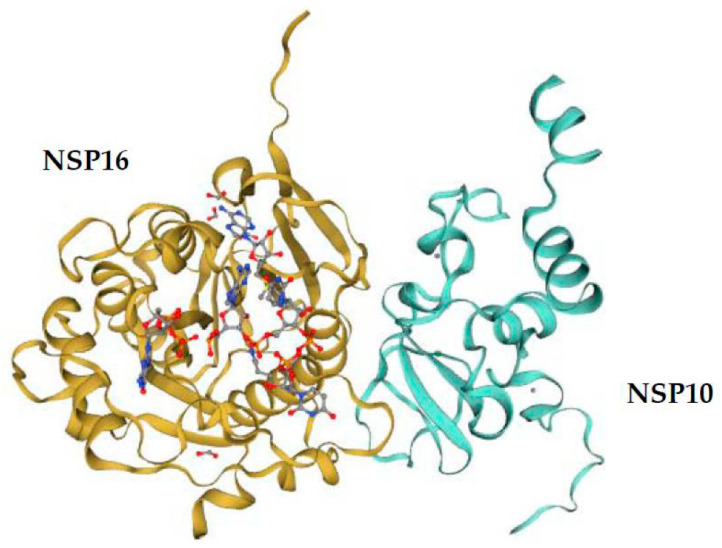
Overall fold of the SARS-CoV-2 NSP10–NSP16 complex. NSP10 is shown in turquoise and NSP16 in gold. PDB ID: 7jyy.1.

In heterodimeric complexes, NSP10 is essential by acting as a cofactor for NSP16 methylase [50,52,53,60,65]. Previous research also confirmed that SARS-CoV-2 NSP10 is highly conserved in SARS-CoV [38]. The NSP10 of SARS-CoV-2 and SARS-CoV consist of 139 residues, and other human coronaviruses’ NSP16 proteins have 135–140 residues. Furthermore, the amino acid sequences of NSP16 and NSP10 are highly conserved in coronaviruses, indicating structural domains and enzymatic functions conserved [66]. The structure of NSP10 is composed of a pair of antiparallel β-strands in the center (Figure 4): one side of the β-strands is surrounded by a crossed large ring, three α-helices, and two 310-residue helices that form two zinc fingers [65,67]. In the aligned human coronaviruses’ NSP10 sequences, the Zn-binding site 1 (coordinating residues C74, C77, H83, and C90) and Zn-binding site 2 (C117, C120, C128, and C130) are 100% conserved (Figure 3). Thus, zinc binding is important for NSP10 activity during the coronavirus replication and transcription processes.

**Figure 5:**
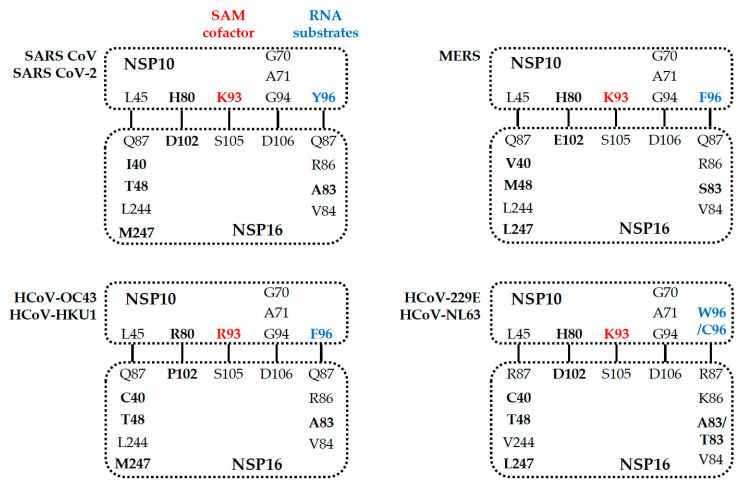
Residues of human coronaviruses involved in the direct interaction of NSP16 and NSP10.

The structure of 2′-O-MTase reveals the critical residues involved in the interaction between NSP10 and NSP16 which might contribute to the binding of cofactor SAM (such as K93 of NSP10 with S105 of NSP16) in SARS-CoV [52], SARS-CoV-2, MERS-CoV, HCoV-229E, and HCoV-NL63 (Figure 5), and RNA substrates (such as Y96 of NSP10 with Q87, R86, A83, and V84 of NSP16) in SARS-CoV [50,52,68] and MERS-CoV. Furthermore, residues N43, Y47, G71, A72, S74, G81, D99, N101, L100, D114, and M131 are coordinated SAM substrate binding sites in SARS-CoV [52], SARS-CoV-2 [67], MERS-CoV, and HCoV-HKU1 through hydrogen bonds and water-mediated interactions. These residues and neighboring water molecules form a hydrogen bond network to expand the conformation to capture the bound SAM [52,67]. Overall, the conservation of the NSP16/NSP10 complex indicates that mutation ablation or alteration of activity would be conserved in the entire virus family, resulting in similar phenotypic mutants [56]. The conservative SAM interaction pattern also highlights the possibility of developing pan-antiviral inhibitors by targeting the SAM binding pocket.

## 6. Coronavirus NSP16-Related Potential Vaccine

A more robust replication induced early during coronavirus infection may improve overall immune performance [64,69]. Enhancing the augmented IFN1 induction via Mda5 can lead to greater TH1 deflection, thereby improving the protective adaptive response. Moreover, the decrease in neutrophils and the increase in the alternatively activated macrophage populations indicate changes in T-cell composition and may affect the immune memory response [70]. Thus, targeting 2′-O-MTase activity via vaccine may serve dual roles of both attenuating the virus and improving the overall immune response.

The mutual regulation of NSP10 and NSP16 maintains 2′-O-MTase activity. The critical KDKE motif is highly conserved, which can predict the functional disruption of the 2′-O-MTase mutation of NSP16 [56]. For example, the NSP16 mutant viruses have aspartic acid (D) mutations in SARS-CoV (D130A), MERS-CoV (D130A), and MHV (D129A) [41,57,64], resulting in mutant KDKE motif viruses that maintain no replication attenuation and a lack of INF1 response [41,57,64]. NSP10 has functional interchangeability in the activation and stimulation of noncognate NSP16 among different coronaviruses. The sequence of the interaction interface of mouse hepatitis virus NSP10, the TP29 peptide, can inhibit the 2′-O-MTase activity of different coronaviruses [46]. In addition, two short peptides from the interaction domains of SARS-CoV NSP10 have been shown to ablate NSP16 2′-O-MTase activity [71]. Thus, by changing the structure and activity of 2′-O-MTase, the mRNA of coronaviruses becomes susceptible to destruction by MDA5 or IFIT1, thereby reducing infection through the natural innate immune response.

Therefore, to change the 2′-O-MTase activity of coronaviruses, candidate vaccines such as attenuated vaccines have been developed. Previous research confirmed that the dNSP16 mutant (D130A) MERS-CoV had a type I interferon-based attenuation and had a protective effect upon challenging with a mouse-adapted MERS-CoV strain [57]. The NSP16 mutant of SARS-CoV-vaccinated mice presented no clinical signs of disease and survived the lethal challenge and indicated a very high level of protective antibody (plaque reduction neutralization titers, PRNT50 > 1:1600) [64]. This PRNT50 value represents an improvement over other live attenuated platforms [69,72]. Therefore, for MERS- and SARS-CoV, the NSP16 mutant viruses can induce a strong protection following lethal homologous challenge in vivo [57,64,73]. Although live attenuated SARS-CoV platforms have had greater efficacy [74,75], there are still several problems. Despite the success of the coronavirus NSP16 mutation in standard young mouse models, it has a significant risk of pathogenesis in aging hosts and has the potential for reversion of virulence [73]. Additionally, a mutation in NSP16 resulting in infectious virus is quite likely since by affecting interferon and the host immune response there would be a selective advantage for the mutated virus to propagate. Finally, NSP16 vaccination must evaluate efficacy against heterologous challenge, aged models of disease, and the potential for reversion, which represent critical factors that must be examined [76,77].

## 7. Drugs Targeting Coronavirus NSP16 Activity

As highlighted earlier, NSP16 needs to be combined with NSP10 to maintain stability and 2′-O-MTase activity [53]. Based on the structural similarity of coronavirus NSP16 with nucleoside analogs, the simulated inhibition mechanism and binding mode can be used for the development of potential therapeutic drugs, a cocktail of anti-coronavirus drugs, broad-spectrum antiviral drugs, or similar or candidate drugs, which have proven effective in clinical trials [78]. Thus, the development of molecules to inhibit cap 2′-O methylation and restore host antiviral response is one approach. The NSP16 2′-O-MTase activity is sensitive to known MTase inhibitors, such as sinefungin and cap analogues [79]. Several candidate drugs have also been identified for their ability to inhibit the activity of coronavirus NSP16, including S-adenosyl-L-homocysteine, sinefungin, and aurintricarboxylic acid [50,53,80]. Recently, using molecular dynamics simulation studies, seven compounds—namely, DB02498, DB03909, DB03186, galuteolin, ZINC000029416466, ZINC000026985532, and ZINC000085537017—have been identified to have an inhibitory effect on SARS-CoV-2 NSP16 [81]. However, pretreatment with sinefungin alone ineffectively reduces SARS-CoV replication. In contrast, in IFN1 induction, the addition of sinefungin improved the overall inhibitory effect [71]. Nonetheless, these candidate drugs, all believed to interfere with substrate binding, effectively ablated 2′-O-MTase activity of both NSP16/NSP10 and NSP14 [53].

In addition to targeting the 2′-O-MTase active site, other approaches also utilize indirect factors necessary for coronavirus NSP16 activity. The specificity of the 2′-O-MTase active site is one of the potential methods, and can significantly impact viral infection with minimal toxicity [78]. Coronavirus NSP16 is a SAM-dependent 2′-O-MTase, which is thought to methylate the ribose 2′-OH of the first transcribed nucleotide of viral RNA cap structures [62]. Thus, the development of molecules to inhibit cap 2′-O methylation and restore the host antiviral response is yet another approach. Compared with the ligands of NSP16-interacting residues, carbamate nicotinamide adenine dinucleotide (DB02498) and galactolignin have inhibitory effects [81]. Therefore, drugs that interfere with this interaction may prove to be effective treatment strategies for SARS-CoV-2 and other coronaviruses.

## 8. Conclusions

Over the past few centuries, coronaviruses have repeatedly emerged from zoonotic reservoirs and are important emerging human pathogens. The complex interaction between NSP16 and NSP10 is necessary for their stability and 2′-O-MTase activity. In the absence of functional NSP16, enhanced recognition of viral RNA by host sensor molecules occurs, such as by the IFIT family, which cause significant attenuation of infections. Notably, the KDKE motif mutation of NSP16 can change the activity of 2′-O-MTase. The targeted SAM-binding site is also expected to interact with 2′-O-MTase encoded by other viruses, thereby providing a wide range of antiviral therapeutics. Importantly, SARS-CoV-2 mutant strains continue to appear, thus the need for coronavirus prevention and treatment methods towards the highly conserved NSP16 activity.

## Figures and Tables

**Table 1:** Comparison of SARS-CoV-2 NSP16/NSP10 amino acid composition with that of other human coronaviruses.

		SARS-CoV-2	SARS-CoV	MERS-CoV	HCoV-OC43	HCoV-HKU1	HCoV-229E	HCoV-NL63
		*Betacoronavirus*					*Alphacoronavirus*	
**NSP16**								
Numbers		YP009725311	NP828873	YP009047227	YP009555257	YP460023	NC002645	NC005831
Length (AA)/MW *		298/33,3230.32	298/33,5040.50	303/33,7070.72	299/33,4260.67	299/33,5810.59	298/33,228.06	300/33,693.72
pI ^#^/Charge at pH 7		7.59/1.16	7.82/1.26	6.24/−1.84	5.95/−2.04	5.81/−2.96	6.29/−1.80	6.91/0.12
AA (%) ^§^	Charged	75 (25.17)	78 (26.17)	73 (24.09)	77 (25.75)	78 (26.09)	86 (28.86)	87 (29.00)
	Acidic	26 (8.72)	27 (9.06)	27 (8.91)	26 (8.7)	27 (9.03)	28 (9.4)	28 (9.33)
	Basic	27 (9.06)	28 (9.40)	25 (8.25)	24 (8.03)	24 (8.03)	26 (8.72)	28 (9.33)
	Polar	91 (30.54)	89 (29.87)	95 (31.35)	85 (28.43)	92 (30.77)	93 (31.21)	93 (31.00)
	Hydrophobic	107 (35.91)	107 (35.91)	113 (37.29)	115 (38.46)	110 (36.79)	107 (35.91)	107 (35.67)
**NSP10**								
Numbers		YP009725306	NP828868	YP009047222	YP009555253	YP459939	NC002645	NC005831
Length (AA)/MW *		139/14,7890.92	139/14,8430.98	140/14,8900.93	137/14,5790.61	137/14,6060.89	135/14,395.37	135/14,162.20
pI ^#^/Charge at pH 7		6.29/−1.10	6.30/−1.10	6.88/−0.16	6.30/−1.07	6.30/−1.07	7.66/1.08	7.62/0.98
AA (%) ^§^	Charged	42 (30.22)	42 (30.22)	38 (27.14)	43 (31.39)	42 (30.66)	40 (29.63)	40 (29.63)
	Acidic	11 (7.91)	11 (7.91)	10 (7.14)	13 (9.49)	12 (8.76)	9 (6.67)	9 (6.67)
	Basic	10 (7.19)	10 (7.19)	10 (7.14)	12 (8.76)	11 (8.03)	10 (7.41)	10 (7.41)
	Polar	50 (35.97)	50 (35.97)	51 (36.43)	46 (33.58)	41 (29.93)	50 (37.04)	48 (35.56)
	Hydrophobic	40 (28.78)	39 (28.06)	45 (32.14)	43 (31.39)	49 (35.77)	41 (30.37)	42 (31.11)

AA: amino acid, MW: molecular weight, pI: Isoelectric point. ^#^^:^ Theoretical pI, *: MW (average), ^§^: AA (frequency %), Charged: RKHYCDE, Acidic: DE, Basic: KR, Polar: NCQSTY.

## Data Availability

No new data were created or analyzed in this study. Data sharing is not applicable to this article.

## References

[1] Coronaviridae Study Group of the International Committee on Taxonomy of Viruses (2020). The species Severe acute respiratory syndrome-related coronavirus: Classifying 2019-nCoV and naming it SARS-CoV-2. Nat. Microbiol..

[2] WHO Novel Coronavirus (2019-nCoV) Situation Report 23. https://www.who.int/docs/default-source/searo/indonesia/covid19/external-situation-report-23-02september2020.pdf?sfvrsn=7ed23646_2.

[3] WHO (2005). Statement on the Second Meeting of the International Health Regulations Emergency Committee Regarding the Outbreak of Novel Coronavirus (2019-nCoV). https://www.who.int/news-room/detail/30-01-2020-statement-on-the-second-meeting-of-the-international-health-regulations-(2005)-emergency-committee-regarding-the-outbreak-of-novel-coronavirus-(2019-ncov).

[4] Woo P.C., Lau S.K., Chu C.M., Chan K.H., Tsoi H.W., Huang Y., Wong B.H., Poon R.W., Cai J.J., Luk W.K. (2005). Characterization and complete genome sequence of a novel coronavirus, coronavirus HKU1, from patients with pneumonia. J. Virol..

[5] Lau S.K., Lee P., Tsang A.K., Yip C.C., Tse H., Lee R.A., So L.Y., Lau Y.L., Chan K.H., Woo P.C. (2011). Molecular epidemiology of human coronavirus OC43 reveals evolution of different genotypes over time and recent emergence of a novel genotype due to natural recombination. J. Virol..

[6] Cui J., Li F., Shi Z.L. (2019). Origin and evolution of pathogenic coronaviruses. Nat. Rev. Microbiol..

[7] Wu D., Koganti R., Lambe U.P., Yadavalli T., Nandi S.S., Shukla D. (2020). Vaccines and Therapies in Development for SARS-CoV-2 Infections. J. Clin. Med..

[8] Xie Y., Wang Z., Liao H., Marley G., Wu D., Tang W. (2020). Epidemiologic, clinical, and laboratory findings of the COVID-19 in the current pandemic: Systematic review and meta-analysis. BMC Infect. Dis..

[9] Wang X.S., Cao F., Zhang Y., Pan H.F. (2020). Therapeutic potential of aryl hydrocarbon receptor in autoimmunity. Inflammopharmacology.

[10] Du L., He Y., Zhou Y., Liu S., Zheng B.J., Jiang S. (2009). The spike protein of SARS-CoV—A target for vaccine and therapeutic development. Nat. Rev. Microbiol..

[11] WHO Severe Acute Respiratory Syndrome (SARS). https://www.who.int/health-topics/severe-acute-respiratory-syndrome#tab=tab_1.

[12] De Groot R.J., Baker S.C., Baric R.S., Brown C.S., Drosten C., Enjuanes L., Fouchier R.A., Galiano M., Gorbalenya A.E., Memish Z.A. (2013). Middle East respiratory syndrome coronavirus (MERS-CoV): Announcement of the Coronavirus Study Group. J. Virol..

[13] WHO Middle East Respiratory Syndrome Coronavirus (MERS-CoV). https://applications.emro.who.int/docs/EMROPub-MERS-SEP-2019-EN.pdf?ua=1&ua=1.

[14] WHO WHO Coronavirus Disease (COVID-19) Dashboard. https://covid19.who.int/.

[15] Suryawanshi R.K., Koganti R., Agelidis A., Patil C.D., Shukla D. (2020). Dysregulation of Cell Signaling by SARS-CoV-2. Trends Microbiol..

[16] Li F. (2016). Structure, Function, and Evolution of Coronavirus Spike Proteins. Annu. Rev. Virol..

[17] Subissi L., Posthuma C.C., Collet A., Zevenhoven-Dobbe J.C., Gorbalenya A.E., Decroly E., Snijder E.J., Canard B., Imbert I. (2014). One severe acute respiratory syndrome coronavirus protein complex integrates processive RNA polymerase and exonuclease activities. Proc. Natl. Acad. Sci. USA.

[18] Rota P.A., Oberste M.S., Monroe S.S., Nix W.A., Campagnoli R., Icenogle J.P., Penaranda S., Bankamp B., Maher K., Chen M.H. (2003). Characterization of a novel coronavirus associated with severe acute respiratory syndrome. Science.

[19] Brian D.A., Baric R.S. (2005). Coronavirus genome structure and replication. Curr. Top. Microbiol. Immunol..

[20] Knoops K., Kikkert M., Worm S.H., Zevenhoven-Dobbe J.C., van der Meer Y., Koster A.J., Mommaas A.M., Snijder E.J. (2008). SARS-coronavirus replication is supported by a reticulovesicular network of modified endoplasmic reticulum. PLoS Biol..

[21] van Hemert M.J., van den Worm S.H., Knoops K., Mommaas A.M., Gorbalenya A.E., Snijder E.J. (2008). SARS-coronavirus replication/transcription complexes are membrane-protected and need a host factor for activity in vitro. PLoS Pathog..

[22] Romano M., Ruggiero A., Squeglia F., Maga G., Berisio R. (2020). A Structural View of SARS-CoV-2 RNA Replication Machinery: RNA Synthesis, Proofreading and Final Capping. Cells.

[23] Ma Y., Wu L., Shaw N., Gao Y., Wang J., Sun Y., Lou Z., Yan L., Zhang R., Rao Z. (2015). Structural basis and functional analysis of the SARS coronavirus nsp14-nsp10 complex. Proc. Natl. Acad. Sci. USA.

[24] Durmus S., Ulgen K.O. (2017). Comparative interactomics for virus-human protein-protein interactions: DNA viruses versus RNA viruses. FEBS Open Bio.

[25] Mirza M.U., Froeyen M. (2020). Structural elucidation of SARS-CoV-2 vital proteins: Computational methods reveal potential drug candidates against main protease, Nsp12 polymerase and Nsp13 helicase. J. Pharm. Anal..

[26] Wu C., Chen X., Cai Y., Xia J., Zhou X., Xu S., Huang H., Zhang L., Zhou X., Du C. (2020). Risk Factors Associated with Acute Respiratory Distress Syndrome and Death in Patients With Coronavirus Disease 2019 Pneumonia in Wuhan, China. JAMA Intern. Med..

[27] Fehr A.R., Perlman S. (2015). Coronaviruses: An overview of their replication and pathogenesis. Methods Mol. Biol..

[28] Peiris J.S., Chu C.M., Cheng V.C., Chan K.S., Hung I.F., Poon L.L., Law K.I., Tang B.S., Hon T.Y., Chan C.S. (2003). Clinical progression and viral load in a community outbreak of coronavirus-associated SARS pneumonia: A prospective study. Lancet.

[29] Nassar M.S., Bakhrebah M.A., Meo S.A., Alsuabeyl M.S., Zaher W.A. (2018). Middle East Respiratory Syndrome Coronavirus (MERS-CoV) infection: Epidemiology, pathogenesis and clinical characteristics. Eur. Rev. Med. Pharmacol. Sci..

[30] Xu Z., Shi L., Wang Y., Zhang J., Huang L., Zhang C., Liu S., Zhao P., Liu H., Zhu L. (2020). Pathological findings of COVID-19 associated with acute respiratory distress syndrome. Lancet Respir. Med..

[31] Wu D., Yang X.O. (2020). TH17 responses in cytokine storm of COVID-19: An emerging target of JAK2 inhibitor Fedratinib. J. Microbiol. Immunol. Infect..

[32] Conti P., Ronconi G., Caraffa A., Gallenga C.E., Ross R., Frydas I., Kritas S.K. (2020). Induction of pro-inflammatory cytokines (IL-1 and IL-6) and lung inflammation by Coronavirus-19 (COVI-19 or SARS-CoV-2): Anti-inflammatory strategies. J. Biol. Regul. Homeost. Agents.

[33] Huang C., Wang Y., Li X., Ren L., Zhao J., Hu Y., Zhang L., Fan G., Xu J., Gu X. (2020). Clinical features of patients infected with 2019 novel coronavirus in Wuhan, China. Lancet.

[34] Fang Y., Zhang H., Xu Y., Xie J., Pang P., Ji W. (2020). CT Manifestations of Two Cases of 2019 Novel Coronavirus (2019-nCoV) Pneumonia. Radiology.

[35] Tisoncik J.R., Korth M.J., Simmons C.P., Farrar J., Martin T.R., Katze M.G. (2012). Into the eye of the cytokine storm. Microbiol. Mol. Biol. Rev..

[36] Channappanavar R., Perlman S. (2017). Pathogenic human coronavirus infections: Causes and consequences of cytokine storm and immunopathology. Semin Immunopathol..

[37] Xia H., Cao Z., Xie X., Zhang X., Chen J.Y., Wang H., Menachery V.D., Rajsbaum R., Shi P.Y. (2020). Evasion of Type I Interferon by SARS-CoV-2. Cell Rep..

[38] Lei X., Dong X., Ma R., Wang W., Xiao X., Tian Z., Wang C., Wang Y., Li L., Ren L. (2020). Activation and evasion of type I interferon responses by SARS-CoV-2. Nat. Commun..

[39] Bouvet M., Lugari A., Posthuma C.C., Zevenhoven J.C., Bernard S., Betzi S., Imbert I., Canard B., Guillemot J.C., Lecine P. (2014). Coronavirus Nsp10, a critical co-factor for activation of multiple replicative enzymes. J. Biol. Chem..

[40] Decroly E., Ferron F., Lescar J., Canard B. (2011). Conventional and unconventional mechanisms for capping viral mRNA. Nat. Rev. Microbiol..

[41] Zust R., Cervantes-Barragan L., Habjan M., Maier R., Neuman B.W., Ziebuhr J., Szretter K.J., Baker S.C., Barchet W., Diamond M.S. (2011). Ribose 2′-O-methylation provides a molecular signature for the distinction of self and non-self mRNA dependent on the RNA sensor Mda5. Nat. Immunol..

[42] Pichlmair A., Schulz O., Tan C.P., Naslund T.I., Liljestrom P., Weber F., Reis e Sousa C. (2006). RIG-I-mediated antiviral responses to single-stranded RNA bearing 5’-phosphates. Science.

[43] Hornung V., Ellegast J., Kim S., Brzozka K., Jung A., Kato H., Poeck H., Akira S., Conzelmann K.K., Schlee M. (2006). 5′-Triphosphate RNA is the ligand for RIG-I. Science.

[44] Ray D., Shah A., Tilgner M., Guo Y., Zhao Y., Dong H., Deas T.S., Zhou Y., Li H., Shi P.Y. (2006). West Nile virus 5’-cap structure is formed by sequential guanine N-7 and ribose 2’-O methylations by nonstructural protein 5. J. Virol..

[45] Daffis S., Szretter K.J., Schriewer J., Li J., Youn S., Errett J., Lin T.Y., Schneller S., Zust R., Dong H. (2010). 2′-O methylation of the viral mRNA cap evades host restriction by IFIT family members. Nature.

[46] Wang Y., Sun Y., Wu A., Xu S., Pan R., Zeng C., Jin X., Ge X., Shi Z., Ahola T. (2015). Coronavirus nsp10/nsp16 Methyltransferase Can Be Targeted by nsp10-Derived Peptide In Vitro and In Vivo To Reduce Replication and Pathogenesis. J. Virol..

[47] Kell A.M., Gale M. (2015). RIG-I in RNA virus recognition. Virology.

[48] Pichlmair A., Schulz O., Tan C.P., Rehwinkel J., Kato H., Takeuchi O., Akira S., Way M., Schiavo G., Reis e Sousa C. (2009). Activation of MDA5 requires higher-order RNA structures generated during virus infection. J. Virol..

[49] von Grotthuss M., Wyrwicz L.S., Rychlewski L. (2003). mRNA cap-1 methyltransferase in the SARS genome. Cell.

[50] Decroly E., Debarnot C., Ferron F., Bouvet M., Coutard B., Imbert I., Gluais L., Papageorgiou N., Sharff A., Bricogne G. (2011). Crystal structure and functional analysis of the SARS-coronavirus RNA cap 2’-O-methyltransferase nsp10/nsp16 complex. PLoS Pathog..

[51] Chen Y., Guo D. (2016). Molecular mechanisms of coronavirus RNA capping and methylation. Virol. Sin..

[52] Chen Y., Su C., Ke M., Jin X., Xu L., Zhang Z., Wu A., Sun Y., Yang Z., Tien P. (2011). Biochemical and structural insights into the mechanisms of SARS coronavirus RNA ribose 2’-O-methylation by nsp16/nsp10 protein complex. PLoS Pathog..

[53] Bouvet M., Debarnot C., Imbert I., Selisko B., Snijder E.J., Canard B., Decroly E. (2010). In vitro reconstitution of SARS-coronavirus mRNA cap methylation. PLoS Pathog..

[54] Bouvet M., Ferron F., Imbert I., Gluais L., Selisko B., Coutard B., Canard B., Decroly E. (2012). Capping strategies in RNA viruses. Med. Sci..

[55] Bollati M., Milani M., Mastrangelo E., Ricagno S., Tedeschi G., Nonnis S., Decroly E., Selisko B., de Lamballerie X., Coutard B. (2009). Recognition of RNA cap in the Wesselsbron virus NS5 methyltransferase domain: Implications for RNA-capping mechanisms in Flavivirus. J. Mol. Biol..

[56] Menachery V.D., Debbink K., Baric R.S. (2014). Coronavirus non-structural protein 16: Evasion, attenuation, and possible treatments. Virus Res..

[57] Menachery V.D., Gralinski L.E., Mitchell H.D., Dinnon K.H., Leist S.R., Yount B.L., Graham R.L., McAnarney E.T., Stratton K.G., Cockrell A.S. (2017). Middle East Respiratory Syndrome Coronavirus Nonstructural Protein 16 Is Necessary for Interferon Resistance and Viral Pathogenesis. mSphere.

[58] Almazan F., Dediego M.L., Galan C., Escors D., Alvarez E., Ortego J., Sola I., Zuniga S., Alonso S., Moreno J.L. (2006). Construction of a severe acute respiratory syndrome coronavirus infectious cDNA clone and a replicon to study coronavirus RNA synthesis. J. Virol..

[59] Martin J.L., McMillan F.M. (2002). SAM (dependent) I AM: The S-adenosylmethionine-dependent methyltransferase fold. Curr. Opin Struct. Biol..

[60] Viswanathan T., Arya S., Chan S.H., Qi S., Dai N., Misra A., Park J.G., Oladunni F., Kovalskyy D., Hromas R.A. (2020). Structural basis of RNA cap modification by SARS-CoV-2. Nat. Commun..

[61] Schubert H.L., Blumenthal R.M., Cheng X. (2003). Many paths to methyltransfer: A chronicle of convergence. Trends Biochem. Sci..

[62] Aouadi W., Blanjoie A., Vasseur J.J., Debart F., Canard B., Decroly E. (2017). Binding of the Methyl Donor S-Adenosyl-l-Methionine to Middle East Respiratory Syndrome Coronavirus 2’-O-Methyltransferase nsp16 Promotes Recruitment of the Allosteric Activator nsp10. J. Virol..

[63] Decroly E., Imbert I., Coutard B., Bouvet M., Selisko B., Alvarez K., Gorbalenya A.E., Snijder E.J., Canard B. (2008). Coronavirus nonstructural protein 16 is a cap-0 binding enzyme possessing (nucleoside-2’O)-methyltransferase activity. J. Virol..

[64] Menachery V.D., Yount B.L., Josset L., Gralinski L.E., Scobey T., Agnihothram S., Katze M.G., Baric R.S. (2014). Attenuation and restoration of severe acute respiratory syndrome coronavirus mutant lacking 2’-o-methyltransferase activity. J. Virol..

[65] Lin S., Chen H., Ye F., Chen Z., Yang F., Zheng Y., Cao Y., Qiao J., Yang S., Lu G. (2020). Crystal structure of SARS-CoV-2 nsp10/nsp16 2′-O-methylase and its implication on antiviral drug design. Signal. Transduct. Target. Ther..

[66] Snijder E.J., Bredenbeek P.J., Dobbe J.C., Thiel V., Ziebuhr J., Poon L.L., Guan Y., Rozanov M., Spaan W.J., Gorbalenya A.E. (2003). Unique and conserved features of genome and proteome of SARS-coronavirus, an early split-off from the coronavirus group 2 lineage. J. Mol. Biol..

[67] Rosas-Lemus M., Minasov G., Shuvalova L., Inniss N.L., Kiryukhina O., Wiersum G., Kim Y., Jedrzejczak R., Maltseva N.I., Endres M. (2020). The crystal structure of nsp10-nsp16 heterodimer from SARS-CoV-2 in complex with S-adenosylmethionine. bioRxiv.

[68] Lugari A., Betzi S., Decroly E., Bonnaud E., Hermant A., Guillemot J.C., Debarnot C., Borg J.P., Bouvet M., Canard B. (2010). Molecular mapping of the RNA Cap 2′-O-methyltransferase activation interface between severe acute respiratory syndrome coronavirus nsp10 and nsp16. J. Biol. Chem..

[69] Fett C., DeDiego M.L., Regla-Nava J.A., Enjuanes L., Perlman S. (2013). Complete protection against severe acute respiratory syndrome coronavirus-mediated lethal respiratory disease in aged mice by immunization with a mouse-adapted virus lacking E protein. J. Virol..

[70] Page C., Goicochea L., Matthews K., Zhang Y., Klover P., Holtzman M.J., Hennighausen L., Frieman M. (2012). Induction of alternatively activated macrophages enhances pathogenesis during severe acute respiratory syndrome coronavirus infection. J. Virol..

[71] Ke M., Chen Y., Wu A., Sun Y., Su C., Wu H., Jin X., Tao J., Wang Y., Ma X. (2012). Short peptides derived from the interaction domain of SARS coronavirus nonstructural protein nsp10 can suppress the 2’-O-methyltransferase activity of nsp10/nsp16 complex. Virus Res..

[72] Graham R.L., Becker M.M., Eckerle L.D., Bolles M., Denison M.R., Baric R.S. (2012). A live, impaired-fidelity coronavirus vaccine protects in an aged, immunocompromised mouse model of lethal disease. Nat. Med..

[73] Menachery V.D., Gralinski L.E., Mitchell H.D., Dinnon K.H., Leist S.R., Yount B.L., McAnarney E.T., Graham R.L., Waters K.M., Baric R.S. (2018). Combination Attenuation Offers Strategy for Live Attenuated Coronavirus Vaccines. J. Virol..

[74] Bolles M., Deming D., Long K., Agnihothram S., Whitmore A., Ferris M., Funkhouser W., Gralinski L., Totura A., Heise M. (2011). A double-inactivated severe acute respiratory syndrome coronavirus vaccine provides incomplete protection in mice and induces increased eosinophilic proinflammatory pulmonary response upon challenge. J. Virol..

[75] Sheahan T., Whitmore A., Long K., Ferris M., Rockx B., Funkhouser W., Donaldson E., Gralinski L., Collier M., Heise M. (2011). Successful vaccination strategies that protect aged mice from lethal challenge from influenza virus and heterologous severe acute respiratory syndrome coronavirus. J. Virol..

[76] Ge X.Y., Li J.L., Yang X.L., Chmura A.A., Zhu G., Epstein J.H., Mazet J.K., Hu B., Zhang W., Peng C. (2013). Isolation and characterization of a bat SARS-like coronavirus that uses the ACE2 receptor. Nature.

[77] He B., Zhang Y., Xu L., Yang W., Yang F., Feng Y., Xia L., Zhou J., Zhen W., Feng Y. (2014). Identification of diverse alphacoronaviruses and genomic characterization of a novel severe acute respiratory syndrome-like coronavirus from bats in China. J. Virol..

[78] Vithani N., Ward M.D., Zimmerman M.I., Novak B., Borowsky J.H., Singh S., Bowman G.R. (2020). SARS-CoV-2 Nsp16 activation mechanism and a cryptic pocket with pan-coronavirus antiviral potential. bioRxiv.

[79] Krafcikova P., Silhan J., Nencka R., Boura E. (2020). Structural analysis of the SARS-CoV-2 methyltransferase complex involved in RNA cap creation bound to sinefungin. Nat. Commun..

[80] He R., Adonov A., Traykova-Adonova M., Cao J., Cutts T., Grudesky E., Deschambaul Y., Berry J., Drebot M., Li X. (2004). Potent and selective inhibition of SARS coronavirus replication by aurintricarboxylic acid. Biochem. Biophys. Res. Commun..

[81] Vijayan V., Pant P., Vikram N., Kaur P., Singh T.P., Sharma S., Sharma P. (2020). Identification of promising drug candidates against NSP16 of SARS-CoV-2 through computational drug repurposing study. J. Biomol. Struct. Dyn..

